# A Case of sporadic Creutzfeldt-Jakob disease

**DOI:** 10.1590/0037-8682-0596-2022

**Published:** 2023-02-20

**Authors:** Fatma Şimşek, Recep Yevgi

**Affiliations:** 1 Ataturk University, Faculty of Medicine, Department of Neurology, Erzurum, Turkey.

**FIGURE 1:**
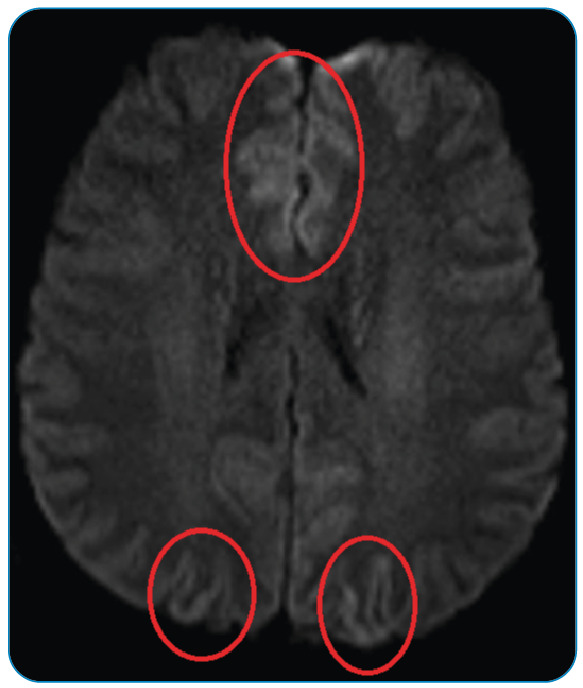
Slightly hyperintense appearances in the cortical gyri of the occipital and frontal lobes on diffusion-weighted MRI

**FIGURE 2:**
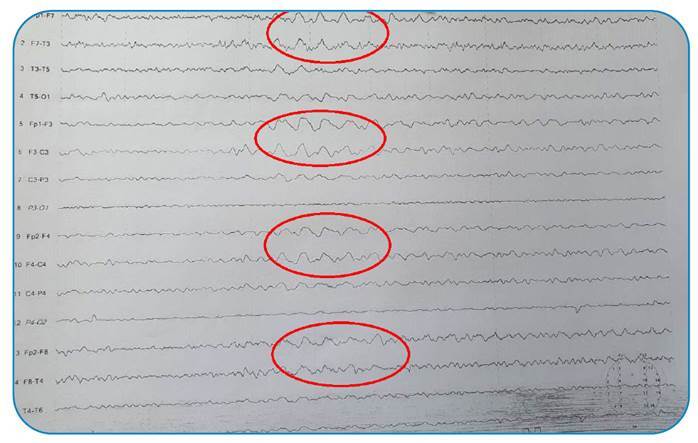
Intermittently repeated slow delta-frequency waves in the frontal regions bilaterally on EEG.

**FIGURE 3:**
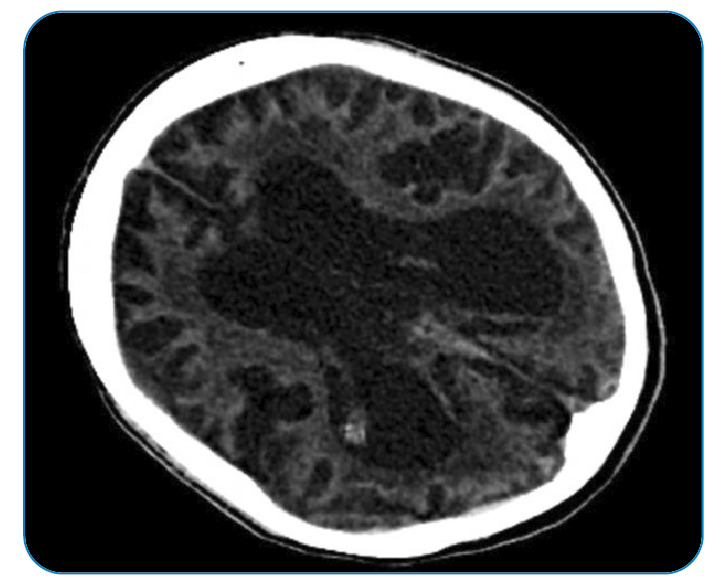
Diffuse cortical atrophy on the brain CT

A 54-year-old male patient with no concomitant disease presented to the Neurology Clinic of Atatürk University Medical Faculty Hospital with complaints of blurred vision and sleeping difficulty for one week. His neurological examination results were normal, except for a visual field defect in the lower quadrant of the right eye. A slightly hyperintense appearance was observed in the cortical gyri of the occipital and frontal lobes on diffusion-weighted magnetic resonance imaging (Figure 1). Long-term electroencephalography (EEG) recordings revealed repetitive slow delta frequency waves, prominent in the frontal regions of both hemispheres without evidence of seizures (Figure 2). One week later, the patient complained of irritability and a startle response. Lumbar puncture revealed no abnormalities in the cerebrospinal fluid biochemistry, microscopic examination, or meningitis panel. The cerebrospinal fluid test results were positive for 14-3-3 protein, a known marker of prion disease. Given the rapid deterioration in the patient’s clinical status, visual complaints, presence of myoclonus, cerebrospinal fluid markers, and EEG findings, a diagnosis of sporadic Creutzfeldt-Jakob disease (CJD) was made. Recurrent bi-frontal delta discharge was observed in continuous EEG recordings of patients with CJD reported in the literature^1^ ^,^ ^2^. A computed tomography (CT) scan of the brain taken one year later revealed a neurodegenerative process and significant cortical atrophy (Figure 3). The patient died shortly after the brain CT. We would like to highlight the possibility of CJD when bi-frontal delta waves are observed in the EEG of patients with acute sleep disturbance.
